# The Impact of b/tsDMARD Dose Reduction on Chronic Hepatitis B in Rheumatoid Arthritis Patients: A Two-Center Long-Term Safety Analysis

**DOI:** 10.3390/jcm12010086

**Published:** 2022-12-22

**Authors:** Der-Yuan Chen, Hsin-Hua Chen, Shih-Hsin Chang, Yi-Ming Chen, Po-Hao Huang, Chia-Wei Hsieh, Joung-Liang Lan, Kuo-Tung Tang

**Affiliations:** 1 Ph.D. Program in Translational Medicine, National Chung Hsing University, Taichung 402, Taiwan; 2 Rheumatology and Immunology Center, China Medical University Hospital, Taichung 404, Taiwan; 3 College of Medicine, China Medical University, Taichung 404, Taiwan; 4 Institute of Medicine, Chung Shan Medical University, Taichung 402, Taiwan; 5 Division of General Medicine, Department of Medicine, Taichung Veterans General Hospital, Taichung 407, Taiwan; 6 Department of Industrial Engineering and Enterprise Information, Tunghai University, Taichung 407, Taiwan; 7 Faculty of Medicine, National Yang Ming Chiao Tung University, Taipei 112, Taiwan; 8 Division of Translational Medicine, Department of Medical Research, Taichung Veterans General Hospital, Taichung 407, Taiwan; 9 Division of Allergy, Immunology and Rheumatology, Taichung Veterans General Hospital, Taichung 407, Taiwan

**Keywords:** antirheumatic agents, biological products, hepatitis B, rheumatoid arthritis

## Abstract

Background: We aimed to investigate the change of hepatitis B virus (HBV) viral loads and HBV reactivation (HBVr) in rheumatoid arthritis (RA) patients after tapering the dose of biological/targeted synthetic disease-modifying antirheumatic drugs (b/tsDMARDs). Methods: This two-center analysis retrospectively investigated the virological and biochemical evidence of HBVr in RA patients who underwent b/tsDMARD dose reduction. Serum levels of viral loads were determined using real-time PCR. Serum levels of alanine transaminase (ALT) were determined using spectrophotometry. Results: Among a total of 40 HBsAg+ RA patients who tapered b/tsDMARDs, 14 (35%) used tocilizumab; 12 (30%) used tumor necrosis factor (TNF)-α inhibitors; and the rest used either abatacept or tofacitinib. We found that patients who had detectable HBV DNA before tapering achieved a one-log reduction in HBV DNA levels, in contrast to the findings in the other 12 patients who did not taper b/tsDMARDs (no change in HBV DNA levels with time). The incidence of HBVr (increased viral loads with hepatitis) was 4.62 (95%CI: 2.08, 10.28) and 2.26 (95%CI: 0.56, 9.02) events per 100 person-years before and after b/tsDMARD tapering, respectively. Conclusions: The HBV viral load decreased after the tapering of b/tsDMARDs in RA patients with detectable HBV DNA. Dose reduction in b/tsDMARDs might be beneficial.

## 1. Introduction

Rheumatoid arthritis (RA) is an inflammatory articular disease characterized by chronic synovitis and bone erosions [1]. The biologic/targeted synthetic disease-modifying antirheumatic drugs (b/tsDMARDs) are effective in RA treatment [1]. For patients who have already achieved clinical remission, a well-recognized treat-to-target goal, or low disease activity (LDA), dose reduction in b/tsDMARDs should be considered to alleviate dose-dependent adverse effects or economic burdens [2,3]. Although disease activity may flare upon discontinuation of b/tsDMARDs [4], proper dose tapering after attainment of remission or LDA allows for sustained positive outcomes [5]. Therefore, the Taiwan Health Insurance Bureau put forward a dose-reducing policy on b/tsDMARD reimbursement since April 2013, recommending dose reduction for patients with remission or LDA after at least two years of therapy. The effect of b/tsDMARD dose reduction on drug safety in RA patients, particularly hepatitis B virus (HBV) reactivation (HBVr), is worthy of investigation, but remains unexplored.

HBV infection is an important public health issue, and more than two billion people have been infected by HBV worldwide [6], 75% of whom live in Southeast Asia and the Western Pacific regions [7]. Taiwan is an HBV-endemic area, with a 7–14% carrier rate of HBV surface antigen (HBsAg) [8,9]. For RA patients with coexisting HBV infection, b/tsDMARD therapy would increase HBVr risk, a common concern particularly in an HBV endemic area [10,11,12,13]. Because HBVr is preventable, guidelines for its prevention, including HBV screening and antiviral prophylaxis, have been proposed by hepatology associations worldwide [14,15,16].

Tumor necrosis factor (TNF)-α suppresses HBV replication and plays a crucial role in eradicating HBV by stimulating HBV-specific cytotoxic T-cell response [13,14,15]. HBVr is a well-known complication in HBV-infected RA patients receiving anti-TNF-α therapy, characterized by the reappearance or rise of HBV DNA in their sera and often associated with hepatocellular injury [16,17,18,19]. Tocilizumab, a monoclonal antibody against IL-6 receptor, is also effective in RA treatment [20], but there is limited data on HBVr in tocilizumab-treated patients [21]. Abatacept binds T cells’ CD80/CD86 costimulatory pathway, thus blocking T-cell activation. Kim et al. reported that HBVr developed in all four abatacept-treated patients who did not receive anti-viral prophylaxis [22], while none of the 38 patients developed HBVr in Padovan’s study [23]. Tofacitinib suppresses multiple key cytokines by inhibiting Janus kinase and alleviates RA symptoms. Chen et al. reported HBVr in two out of four RA patients with chronic hepatitis B (CHB) and received tofacitinib therapy without anti-viral prophylaxis [24]. Other than these findings, the data regarding the influence of b/tsDMARD dose reduction on HBVr risk in RA patients remain scarce.

In this retrospective observational study with a long-term follow-up period between 2006 and 2021, we aimed to investigate the impact of b/tsDMARD dose reduction on HBV viral load and the risk of HBVr in RA patients who had HBV infection.

## 2. Materials and Methods

### 2.1. Patients

In this two-center, long-term analysis from January 2006 to December 2021, we retrospectively reviewed the medical records of 2626 consecutive patients who fulfilled the 1987 revised criteria of the American College of Rheumatology (ACR) [25] or the 2010 classification criteria of the ACR/European League Against Rheumatism (EULAR) collaborative initiative for RA [26]. All patients received b/tsDMARDs based on the guidelines of the British Society for Rheumatology [27]. According to the Taiwan Health Insurance’s dose-reducing policy, implemented in April 2013, the dosage of b/tsDMARDs should be reduced in RA patients who have achieved remission or LDA after receiving therapy for at least 2 years. Those patients who successfully achieved long-term tapering of b/tsDMARDs were selected. In addition, patients who did not taper b/tsDMARDs after a 2-year use of b/tsDMARDs at the Taichung Veterans General Hospital were identified as the control group to investigate the natural history regarding HBV viral loads in the RA population. This study complied with the Declaration of Helsinki, and was approved by the Institutional Review Board of both medical centers (TCVGH CE22031B, CMUH110-REC2-106-AR1). As patient data were anonymized before analysis, the written consent was waived.

### 2.2. Definitions

All participants underwent regular checks for liver enzymes every 3 months and HBV viral load annually since the start of b/tsDMARD therapy. Among the 40 RA patients who tapered b/tsDMARDs, the follow-up period before b/tsDMARD tapering was from their first prescription through the dose tapering. The follow-up period after b/tsDMARD tapering was from the start of dose tapering until either one of the three conditions: dose titration of b/tsDMARDs, discontinuation of b/tsDMARDs, or the end of follow-up. HBVr was defined as follows: HBV DNA turning detectable when it was previously undetectable or a 10-fold rise of HBV DNA level, which was accompanied by the occurrence of hepatitis [28,29,30]. Hepatitis was defined as a 3-fold or more increase in alanine transaminase (ALT) that exceeded the upper limit of normal (44 U/L) or an absolute increase in ALT to more than 120 U/L.

### 2.3. Laboratory Examinations

HBsAg was determined using an electrochemiluminescence immunoassay (Roche Diagnostics, Mannheim, Germany). Serum HBV DNA was extracted using a High Pure Viral Nucleic Acid kit (Roche, Mannheim, Germany), and the viral loads were quantified using a Roche Cobas TaqMan HBV Test (Roche Diagnostics, Basel, Switzerland), with a detection limit of 20 IU/mL. Serum levels of ALT were determined using spectrophotometry (Fujifilm, Osaka, Japan).

### 2.4. Statistical Analysis

The results are presented as the median plus the interquartile range (IQR) unless specified otherwise. The Mann–Whitney U test and chi-squared test were used for between-subject comparisons. The Wilcoxon signed rank test and McNemar’s test were used for within-subject comparisons. The incidences of HBVr were calculated before and after b/tsDMARD tapering, and compared in the random effects Poisson regression. Subgroup analyses were undertaken in patients with detectable HBV DNA levels before b/tsDMARD dose tapering, patients whose status of antiviral drugs use was consistent (patients who used antiviral drugs before and after b/tsDMARD tapering, and patients who did not use antiviral drugs before and after b/tsDMARD tapering), patients who received different b/tsDMARDs, and patients with percentage decreases in b/tsDMARD dosages equal to or more than 50%. In the sensitivity analysis, the effect of institutional difference was accounted for by adding the institution as a level factor in the regression model. A two-side *p* value < 0.05 was considered statistically significant.

## 3. Results

### 3.1. Patients

**Figure 1:**
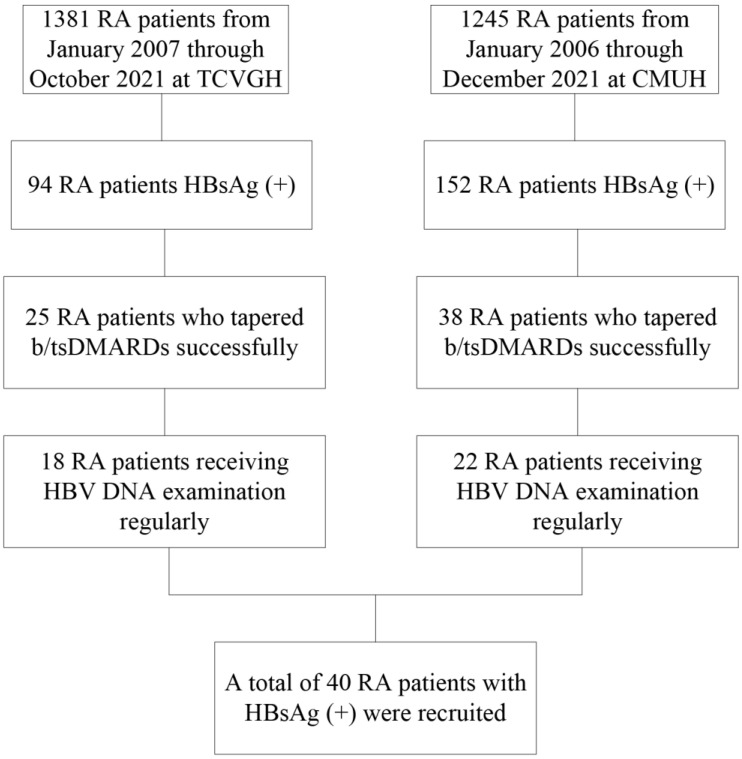
Study flow chart. b/tsDMARDs, biologic/targeted synthetic disease-modifying antirheumatic drugs; CMUH, China Medical University Hospital; DNA, deoxyribonucleic acid; HBV, hepatitis B virus; HBsAg, hepatitis B virus surface antigen; RA, rheumatoid arthritis; TCVGH, Taichung Veterans General Hospital.

The process of patient enrollment is illustrated in Figure 1. We reviewed a total of 2626 patients with RA at the China Medical University Hospital and the Taichung Veterans General Hospital, and 246 (9.4%) had positive HBsAg, which represents concomitant CHB. Sixty-three (26%) patients had successfully tapered b/tsDMARDs. We finally enrolled 40 patients (37 females and 3 males) who had received regular HBV DNA examinations. A total of 12 patients (11 females and one male) who did not taper b/tsDMARDs after 2 years of b/tsDMARDs were also identified.

The clinical characteristics before b/tsDMARD use of the 40 RA patients who tapered b/tsDMARDs are demonstrated in Table 1. Their median age was 54 years. All patients had been treated with methotrexate (MTX) or other conventional synthetic DMARDs (csDMARDs), but still had active disease status (mean DAS28, 6.4; range 3.4–8.0) before the b/tsDMARD therapy was initiated. Fourteen (35%) of them received tocilizumab treatment; twelve (30%) received TNF-α inhibitors (each of etanercept, adalimumab, and golimumab was used in four patients); the rest of them received either abatacept or tofacitinib. The dosages were as follows: etanercept 25 mg twice weekly, adalimumab 40 mg every other week, golimumab 50 mg every month, tocilizumab 4 mg/kg once monthly during the first 3 months and then 8 mg/kg once monthly afterward, abatacept 750 mg every month, and tofacitinib 5 mg twice daily. On average, b/tsDMARDs were reduced by 30% in these patients.

In terms of the control group, they had higher proportions of seropositivity (rheumatoid factor, *p* = 0.04), and a lower disease activity (DAS28-ESR, *p* = 0.03) before b/tsDMARD use when compared with those patients who successfully tapered b/tsDMARDs (Table 1). Half (50%) of them received tocilizumab and the rest of them received TNF-α inhibitors, abatacept, or tofacitinib.

### 3.2. Characteristics before and after b/tsDMARD Dose Tapering

As shown in Table 2, the median follow-up period was 2.5 and 1.2 years before and after the start of b/tsDMARD dose tapering, respectively. The proportions of concomitant use of csDMARDs or preemptive antiviral drugs were not significantly different before and after b/tsDMARD tapering. Only a third of the patients received preemptive antivirals, mostly entecavir. The emergence of HBVr was found in six and two patients before and after b/tsDMARD tapering, respectively.

As for the control group, the proportions of concomitant use of csDMARDs or preemptive antiviral drugs were not significantly different between baseline (defined as 2 years since the first prescription of b/tsDMARDs) and 1 year later (Table S1).

### 3.3. The Change in HBV Viral Loads after b/tsDMARD Dose Tapering

**Figure 2:**
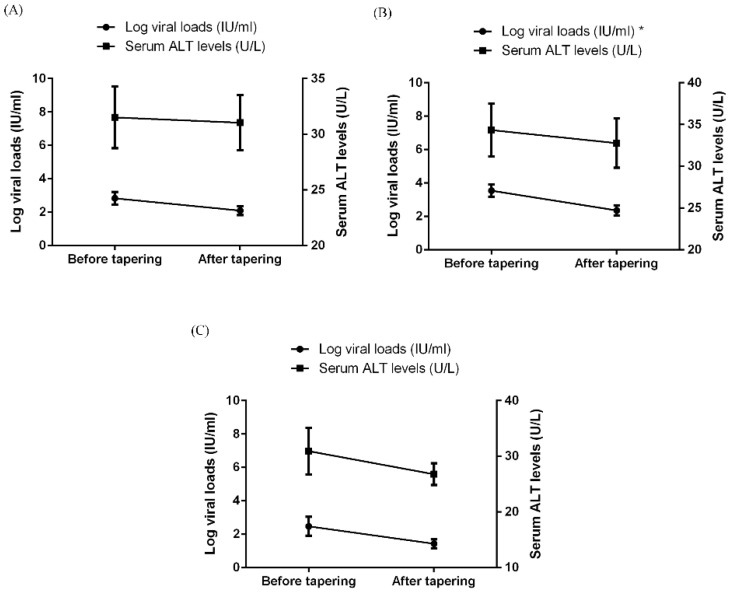
The change in serum levels of viral loads and ALT in RA patients with chronic hepatitis B before and after tapering of b/tsDMARDs, with respect to (**A**) all 40 patients, (**B**) 32 patients with detectable HBV DNA levels before tapering, and (**C**) 14 patients who used tocilizumab. Data are presented as mean ± SEM. * *p* < 0.05. ALT, alanine aminotransferase; b/tsDMARDs, biological/targeted synthetic disease-modifying antirheumatic drugs; HBV, hepatitis B virus; RA, rheumatoid arthritis; SEM, standard error of mean.

As shown in Figure 2A, HBV viral loads were decreased after b/tsDMARD dose tapering, although the statistical significance was not reached (*p* = 0.143). In the subgroup analyses (Figure 2B,C and Figure S1), b/tsDMARD dose tapering was significantly associated with a one-log reduction in HBV viral loads among those with detectable HBV DNA before tapering. There was also a trend toward HBV viral load reduction in those receiving a tapered dose of tocilizumab (*p* = 0.096). Nevertheless, serum ALT levels did not significantly change after dose tapering of any b/tsDMARDs. On the other hand, there was no change in HBV viral loads or serum ALT levels with time in the control group (Figure S2).

### 3.4. The Incidences of HBVr before and after b/tsDMARD Dose Tapering

The incidences of HBVr were calculated to be 4.62 (95%CI: 2.08, 10.28) and 2.26 (95%CI: 0.56, 9.02) per 100 person-years before and after b/tsDMARD tapering (Table 2), respectively. In the random effects Poisson regression, b/tsDMARD dose tapering was not significantly associated with a decrease in HBVr incidence (Table S2). The result was similar when the institutional effect was accounted for in the analysis.

### 3.5. Potential Risk Factors for HBVr in RA Patients before b/tsDMARDs Dose Tapering

To explore the potential risk factors for HBVr in RA patients, we compared clinical characteristics between patients who developed HBVr and those who did not, before b/tsDMARD dose tapering (Table 3). RA patients who developed HBVr had higher CRP levels and a trend toward higher DAS28-ESR (*p* = 0.06) before b/tsDMARD use than those patients who did not.

## 4. Discussion

HBVr, a frequent complication in HBsAg-positive patients, occurrs in 21–53% of HBV carriers receiving cytotoxic agents or immunosuppressants and may lead to acute hepatitis and even hepatic failure [29]. HBVr risk is thought to be high in HBsAg-positive RA patients in Taiwan, where HBV is endemic [11]. The present study is the first attempt to investigate the impact of b/tsDMARD dose tapering on HBV in RA patients. Our results showed that patients with detectable HBV DNA before b/tsDMARD tapering had a 10-fold decrease in HBV viral loads within 11 (IQR, 6, 13; data not shown) months after tapering. The incidence of HBVr decreased after b/tsDMARD dose tapering, although the statistical significance was not reached.

In the present study, the prevalence of HBsAg positivity was 9.4%, similar to that in the Taiwanese general population [9]. One fourth of our RA patients with CHB successfully tapered b/tsDMARDs, and the proportion was seemingly lower than those reported in the previous studies of general RA patients (44–79%) [31]. Nevertheless, the b/tsDMARD tapering strategies and outcome definitions differed between these studies and our real-world observations, making it difficult to make direct comparisons. The amount and duration of dosage reduction with respect to b/tsDMARDs varied at the physician’s discretion given that the dose re-titration of b/tsDMARDs was not protocol-based in our clinical practice.

The treat-to-target approach incorporating b/tsDMARD therapy greatly improved disease control, joint destruction, disability, quality of life, and mortality in RA patients [32,33,34]. Despite inconsistent findings [23,35], HBVr has been observed in RA patients with concomitant CHB after receiving b/tsDMARDs, such as anti-TNF-α therapy [11], abatacept [22], tocilizumab [21,36], and tofacitinib [24]. Similarly, some of our RA patients receiving these b/tsDMARDs developed HBVr. Previous studies suggested a beneficial effect of antiviral prophylaxis on HBVr in RA patients receiving b/tsDMARDs [11,22,24]. In the present study, interestingly, we found that patients who developed HBVr had a higher disease activity before b/tsDMARD use than those patients who did not, despite a similar medication use. Our observation is in line with previous studies showing an increased risk for infection, perhaps due to a relatively immunocompromised status, in RA patients with a higher disease activity [37,38].

Dose reduction in b/tsDMARDs after achieving disease remission or LDA has potential benefits, such as safety improvement and a lighter economic burden [39]. This two-center cohort revealed a one-log reduction in HBV viral loads after tapering b/tsDMARDs in patients with detectable HBV DNA before tapering, which was not observed in the control group. Among the different b/tsDMARDs, there was a trend of HBV viral load reduction in RA patients receiving a tapered dose of tocilizumab. Given that HBV viral loads could predict the development of liver cirrhosis and hepatocellular carcinoma [40,41], b/tsDMARD tapering may help lower the incidence of these complications. Nevertheless, the prognostic value of a one-log reduction in HBV DNA levels in such RA patients is unknown. A future study with a larger sample size and longer follow-up is needed.

Another important sequela of HBVr is resultant hepatitis, which can lead to severe complications including fulminant hepatic failure and even death [42]. In the present study, we noted a lower incidence of HBVr (increased viral loads and accompanying hepatitis) after b/tsDMARD dose tapering, although the event number was insufficient to detect a significant difference. Notably, there was no occurrence of HBVr during a follow-up of 2.4 (SD 2.1) years after dose tapering in patients who received anti-TNF-α therapy, abatacept, or tocilizumab (data not shown). On the contrary, the incidence of HBVr was increased in those who had dose tapering of tofacitinib. The reason for such observations with different b/tsDMARDs needs to be elucidated. Kupffer cells, liver-resident macrophages, secret IL-6 to control HBV infection [43]. IL-6 signaling could be suppressed by tofacitinib and, as a result, HBVr develops. Nonetheless, Janus kinase 1 may mediate the wide-ranging effects of X protein of HBV (HBx), one of which is to facilitate HBV infection through the modulation of hepatocyte apoptosis [44]. In addition, Janus kinase 1 also mediates the immunosuppressive effect of IL-10, which is important in HBV persistence [43]. This may, in part, explain the observed paradoxical effect of tapering tofacitinib on HBVr.

Immunosuppressive and chemotherapeutic agents predispose CHB patients to the development of HBVr due to the loss of immune control [10,45], and the use of corticosteroids, methotrexate, and azathioprine all probably poses some risk for HBVr. In the present study, we showed no significant differences in the use of these medications among the RA patients before and after b/tsDMARD dose tapering. While antiviral drugs could remarkably alleviate HBVr risk in such patients [11], there were no significant differences in the proportion of antiviral drug use by our RA patients before and after b/tsDMARD tapering. Our results remained the same after the use of antiviral drugs were accounted for in the subgroup and regression analysis.

Despite the novel findings presented herein, there are some limitations. Firstly, our study is retrospective in design, and some patients who did not receive regular monitoring of HBV viral loads were excluded. Secondly, the sample size was small and the HBVr events were few, since only a small proportion of RA patients can successfully achieve long-term tapering of b/tsDMARDs [31]. It is therefore difficult to analyze the results regarding different b/tsDMARDs. Thirdly, HBV is endemic in Taiwan and our patients were all Han Chinese; our observations may not be extrapolated to other countries or ethnicities. A prospective international multicenter study is needed to validate our findings.

## 5. Conclusions

In conclusion, HBV viral loads were decreased after b/tsDMARD dose reduction in RA patients with detectable HBV DNA before tapering. There is still debate on whether and how to taper b/tsDMARDs in RA patients [46]. Our findings suggested that RA patients with CHB might benefit from b/tsDMARD dose tapering.

## Figures and Tables

**Table 1:** Characteristics of RA patients before the use of b/tsDMARDs.

Characteristics	RA Patients with CHB Who Successfully Tapered b/tsDMARDs (*n* = 40)	RA Patients with CHB Who Did Not Taper b/tsDMARDs (*n* = 12)	*p* Value
Age (years), median (IQR)	54 (47, 63)	59 (51, 64)	0.39
Female sex, n (%)	37 (93)	11 (92)	0.92
Disease duration (years), median (IQR)	7.5 (3.5, 9.0)	7.5 (5.0, 11.5)	0.29
RF positivity, n (%)	29 (73)	12 (100)	0.04 *
ACPA positivity, n (%) ^a^	29 (74)	9 (90)	0.29
DAS28-ESR, median (IQR)	6.4 (5.9, 7.1)	5.6 (5.2, 6.6)	0.03 *
ESR (mm/hr), median (IQR)	41 (23, 57)	31 (14, 62)	0.55
CRP (mg/dL), median (IQR) ^b^	1.6 (0.6, 3.1)	0.7 (0.2, 2.4)	0.14
bDMARDs and tsDMARDs, n (%)			0.75
TNF-α inhibitors	12 (30)	2 (17)	
Abatacept	6 (15)	2 (17)	
Tocilizumab	14 (35)	6 (50)	
Tofacitinib	8 (20)	2 (17)	
The percentage decrease in b/tsDMARDs (%), mean (SD)	30 (13)	N.A.	N.A.

ACPA, anti-citrullinated protein antibody; b/tsDMARDs, biologic/targeted syntehtic disease modifying anti-rheumatic drugs; CHB, chronic hepatitis B; CRP, C-reactive protein; DAS28-ESR, disease activity score−28 for rheumatoid arthritis with erythrocyte sedimention rate; ESR, erythrocyte sedimention rate; IQR, interquartile range; N.A., not available; RA, rheumatoid arthritis; RF, rheumatoid factor; SD, standard deviation; TNF, tumor necrosis factor. ^a^ Three patients did not receive ACPA examination. ^b^ One patient did not receive CRP examination. * < 0.05.

**Table 2:** HBV reactivation in the 40 RA patients with CHB before and after tapering b/tsDMARDs.

Characteristics	Before Tapering b/tsDMARDs	After Tapering b/tsDMARDs	*p* Value
Follow-up period (years), median (IQR)	2.5 (1.8, 3.1)	1.2 (0.9, 3.2)	0.04 *
Corticosteroids (mg/day), median (IQR) ^a^	2.5 (2.5, 5.0)	2.5 (2.5, 5.0)	0.17
csDMARDs			
Methotrexate (mg/week), median (IQR)	0.0 (0.0, 7.5)	0.0 (0.0, 7.5)	0.16
Cyclosporine, n (%)	4 (10)	4 (10)	N.E.
Salazopyrine, n (%)	8 (20)	8 (20)	N.E.
Azathioprine, n (%)	1 (3)	2 (5)	0.32
Leflunomide, n (%)	5 (13)	5 (13)	N.E.
Hydroxychloroquine, n (%)	26 (65)	27 (68)	0.32
Antiviral drugs for HBV, n (%)	9 (23)	12 (30)	0.26
Entecavir, n (%)	5 (56)	9 (75)	
Lamivudine, n (%)	3 (33)	2 (17)	
Telbivudine, n (%)	1 (11)	1 (8)	
HBV reactivation	6	2	N.A.
Incidence of HBV reactivation (events per 100 person-years [95%CI] ^b^)			
Overall	4.62 (2.08, 10.28)	2.26 (0.56, 9.02)	N.A.
Users of TNF-α inhibitors	4.62 (1.16, 18.48)	0 (0, 0)	N.A.
Abatacept users	8.30 (1.17, 58.90)	0 (0, 0)	N.A.
Tocilizumab users	1.84 (0.26, 13.10)	0 (0, 0)	N.A.
Tofacitinib users	9.84 (2.46, 39.34)	16.20 (4.05, 64.78)	N.A.

ACPA, anti-citrullinated protein antibodies; b/tsDMARDs, biologic/targeted synthetic disease modifying anti-rheumatic drugs; CHB, chronic hepatitis B; csDMARDs, conventional synthetic disease-modifying antirheumatic drugs; HBV, hepatitis B virus; IQR, interquartile range; N.A., not available; N.E., not estimable; RA, rheumatoid arthritis; TNF, tumor necrosis factor. ^a^ presented as prednisone equivalent dose. ^b^ calculated based on the quadratic approximation to the Poisson log likelihood. * < 0.05.

**Table 3:** Characteristics of RA patients who developed HBVr and those who did not before b/tsDMARD dose tapering.

Characteristics	RA Patients Who Developed HBVr (*n* = 6)	RA Patients Who Did Not Develop HBVr (*n* = 34)	*p* Value
Characteristics before b/tsDMARD use			
Age (years), median (IQR)	55 (49, 62)	54 (45, 63)	0.88
Female sex, n (%)	6 (100)	31 (91)	0.45
Disease duration (years), median (IQR)	8.0 (5.0, 8.0)	6.5 (3.0, 10.0)	0.77
RF positivity, n (%)	5 (83)	24 (71)	0.52
ACPA positivity, n (%)^a^	4 (67)	25 (76)	0.64
DAS28-ESR, median (IQR)	7.2 (6.3, 8.0)	6.3 (5.8, 7.0)	0.06
ESR (mm/hr), median (IQR)	50 (26, 75)	40 (22, 55)	0.42
CRP (mg/dL), median (IQR) ^b,^*	3.6 (2.1, 6.4)	1.2 (0.6, 2.9)	0.02 *
Characteristics after b/tsDMARD use			
Follow-up period (years), median (IQR)	2.5 (2.0, 2.7)	2.5 (1.6, 3.3)	0.91
Corticosteroids (mg/day), median (IQR) ^a^	5.0 (2.5, 5.0)	2.5 (2.5, 5.0)	0.62
CsDMARDs			
Methotrexate (mg/week), median (IQR)	0.0 (0.0, 7.5)	0.0 (0.0, 7.5)	0.93
Cyclosporine, n (%)	1 (17)	3 (9)	0.55
Salazopyrine, n (%)	0 (0)	8 (24)	0.18
Azathioprine, n (%)	0 (0)	1 (3)	0.67
Leflunomide, n (%)	1 (17)	4 (12)	0.74
Hydroxychloroquine, n (%)	5 (83)	21 (62)	0.31
b/tsDMARDs, n (%)			0.72
TNF-α inhibitors	2 (33)	10 (29)	
Abatacept	1 (17)	5 (15)	
Tocilizumab	1 (17)	13 (38)	
Tofacitinib	2 (33)	6 (18)	
Antiviral drugs for HBV, n (%)	2 (33)	7 (21)	0.49

ACPA, anti-citrullinated protein antibody; b/tsDMARDs, biologic/targeted syntehtic disease modifying anti-rheumatic drugs; CHB, chronic hepatitis B; CRP, C-reactive protein; csDMARDs; conventional synthetic disease modifying anti-rheumatic drugs; DAS28-ESR, disease activity score−28 for rheumatoid arthritis with erythrocyte sedimention rate; ESR, erythrocyte sedimention rate; HBV, hepatitis B virus; IQR, interquartile range; n, number of individuals; RA, rheumatoid arthritis; RF, rheumatoid factor; TNF, tumor necrosis factor. ^a^ Three patients did not receive ACPA examination. ^b^ One patient did not receive CRP examination. * < 0.05.

## Data Availability

The data that support the findings of this study are available from the corresponding author upon reasonable request.

## Supplementary Materials

The following supporting information can be downloaded at: https://www.mdpi.com/article/10.3390/jcm12010086/s1, Figure S1. The change in serum levels of viral loads and ALT in RA patients with chronic hepatitis B before and after tapering of b/tsDMARDs; T S2. The change in serum levels of viral loads and ALT with time in RA patients who used b/tsDMARDs for more than 2 years without dose reduction, in regards to (A) all 12 patients, (B) 11 patients with detectable HBV DNA levels at baseline; Figure S2. The change in serum levels of viral loads and ALT with time in RA patients who used b/tsDMARDs for more than 2 years without dose reduction, in regards to (A) all 12 patients, (B) 11 patients with detectable HBV DNA levels at baselinea. Data are presented as mean ± SEM; Table S1: Medications used by RA patients with CHB who did not taper b/tsDMARDs after two years of b/tsDMARDs use; Table S2. The effect of b/tsDMARD tapering on the incidence of HBV reactivation in random effects Poisson regression.
