# Supplementary material for: The Impact of b/tsDMARD Dose Reduction on Chronic Hepatitis B in Rheumatoid Arthritis Patients: A Two-Center Long-Term Safety Analysis
Source: J Clin Med. 2022 Dec 22;12(1):86. doi: 10.3390/jcm12010086 (PMC9821696; doi:10.3390/jcm12010086)
Supplement: Supplementary file 1 [file jcm-12-00086-s001.zip › jcm-2066005-supplementary.pdf]

Journal of
Clinical Medicine

Supplementary Materials

The Impact of b/tsDMARD Dose Reduction on Chronic Hepatitis B in Rheumatoid Arthritis Patients: A Two-Center Long-Term Safety Analysis

Der-Yuan Chen [1,2,3,4], Hsin-Hua Chen [1,4,5,6,7], Shih-Hsin Chang [1,2,3], Yi-Ming Chen [1,7,8], Po-Hao Huang [2,3], Chia-Wei Hsieh [1,9], Joung-Liang Lan [2,3] and Kuo-Tung Tang [1,7,9,*]

[1] Ph.D. Program in Translational Medicine, National Chung Hsing University, Taichung 402, Taiwan
[2] Rheumatology and Immunology Center, China Medical University Hospital, Taichung 404, Taiwan
[3] College of Medicine, China Medical University, Taichung 404, Taiwan
[4] Institute of Medicine, Chung Shan Medical University, Taichung 402, Taiwan
[5] Division of General Medicine, Department of Medicine, Taichung Veterans General Hospital, Taichung 407, Taiwan
[6] Department of Industrial Engineering and Enterprise Information, Tunghai University, Taichung 407, Taiwan
[7] Faculty of Medicine, National Yang Ming Chiao Tung University, Taipei 112, Taiwan
[8] Division of Translational Medicine, Department of Medical Research, Taichung Veterans General Hospital, Taichung 407, Taiwan
[9] Division of Allergy, Immunology and Rheumatology, Taichung Veterans General Hospital, Taichung 407, Taiwan
* Correspondence: dirac1982@vghtc.gov.tw; Tel.: +886-4-23592525 (ext. 3334)

Table S1. Medications used by RA patients with CHB who did not taper b/tsDMARDs after two years of b/tsDMARDs use.

Medications	Baseline	One year later
csDMARDs		
Corticosteroids (mg/day)[a]	4.3 (2.9)	4.7 (3.5)
Methotrexate (mg/week)	3.3 (5.7)	2.9 (4.4)
Cyclosporine, n (%)	0 (0)	0 (0)
Salazopyrine, n (%)	3 (25)	3 (25)
Azathioprine, n (%)	0 (0)	0 (0)
Leflunomide, n (%)	1 (8)	1 (8)
Hydroxychloroquine, n (%)	4 (33)	4 (33)
Antiviral drugs for HBV, n (%)	3 (25)	3 (25)
Entecavir, n (%)	3 (100)	2 (67)
Telbivudine, n (%)	0 (0)	1 (33)

ACPA, anti-citrullinated protein antibodies; b/tsDMARDs, biologic/targeted synthetic disease modifying anti-rheumatic drugs; CHB, chronic hepatitis B; csDMARDs, conventional synthetic disease-modifying antirheumatic drugs; HBV, hepatitis B virus; RA, rheumatoid arthritis; TNF, tumor necrosis factor.

[a]presented as prednisone equivalent dose.

[b]calculated based on the quadratic approximation to the Poisson log likelihood.

Table S2. The effect of b/tsDMARD tapering on the incidence of HBV reactivation in random effects Poisson regression[a].

	IRR (95% CI)
**Primary analysis**	0.50 (0.08, 2.95)
**Stratification analysis**	
Patients with detectable HBV DNA levels before b/tsDMARDs tapering	0.52 (0.09, 3.17)
Patients whose status of antiviral drugs use was consistent before and after b/tsDMARDs tapering	0.79 (0.12, 5.24)
Patients whose percentage decrease in b/tsDMARDs was equal to or more than 50%	N.E.
Users of TNF-α inhibitors	N.E.
Abatacept users	N.E.
Tocilizumab users	N.E.
Tofacitinib users	5.24 (0.57, 47.9)
**Sensitivity analysis**	
Accounting for the institutional effect	0.64 (0.10, 4.09)

b/tsDMARDs, biologic/targeted synthetic disease modifying anti-rheumatic drugs; HBV, hepatitis B; IRR, incidence rate ratio; N.E., not estimable; TNF, tumor necrosis factor.

[a]adjusted for the use of antiviral drugs.

(A)

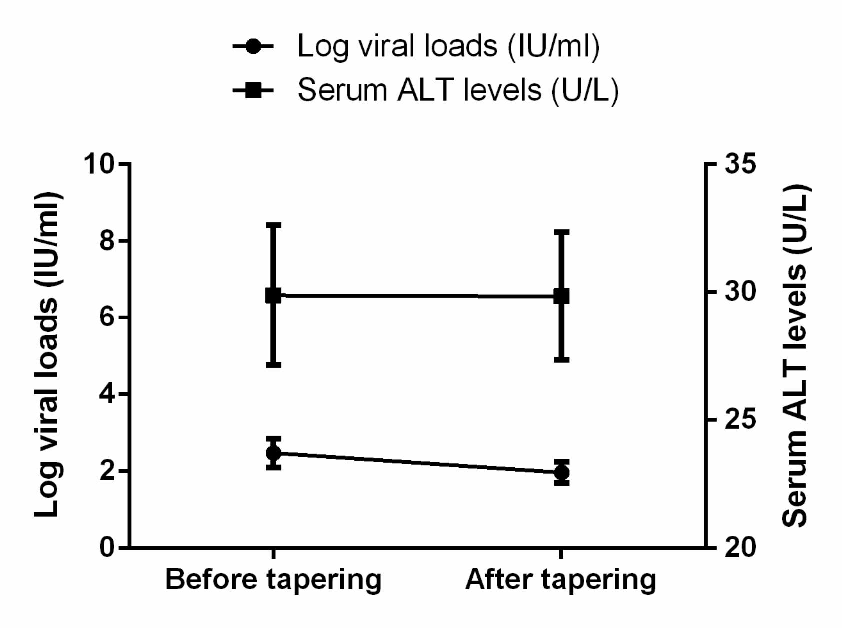

(B)

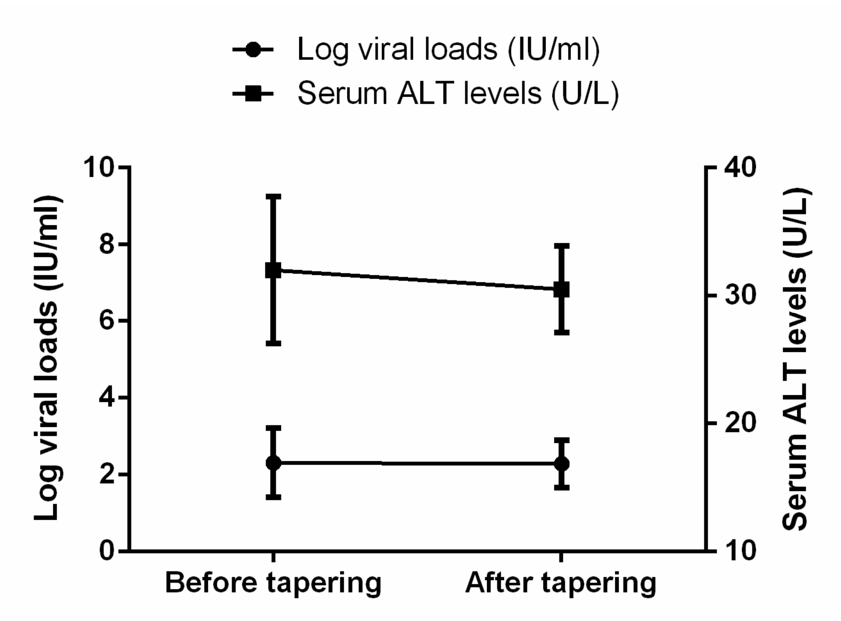
Log viral loads (IU/ml)
Serum ALT levels (U/L)
Log viral loads (IU/ml)
Serum ALT levels (U/L)
10
8
6
4
2
0
40
30
20
10
Before tapering
After tapering

(C)

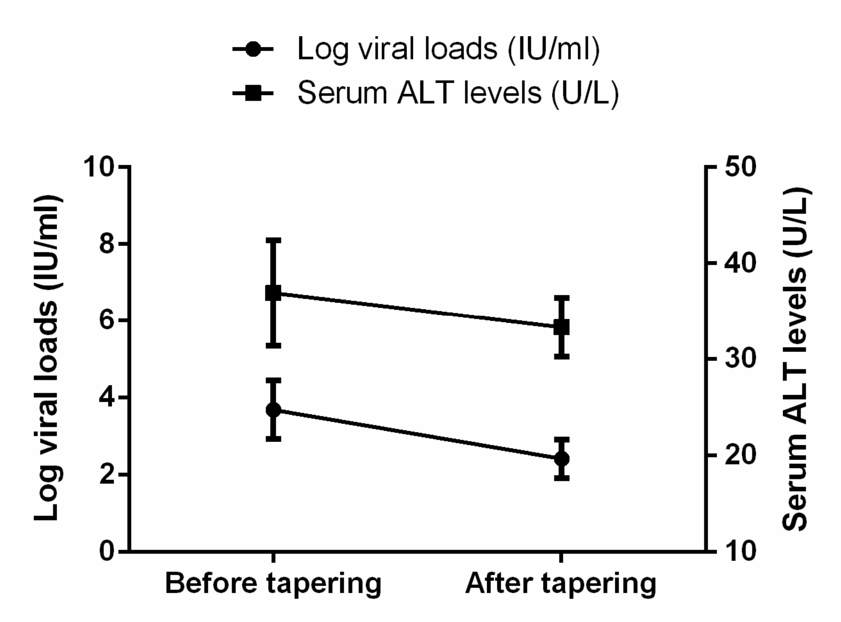
Log viral loads (IU/ml)
Serum ALT levels (U/L)
Log viral loads (IU/ml)
Serum ALT levels (U/L)
10
8
6
4
2
0
50
40
30
20
10
Before tapering
After tapering

(D)

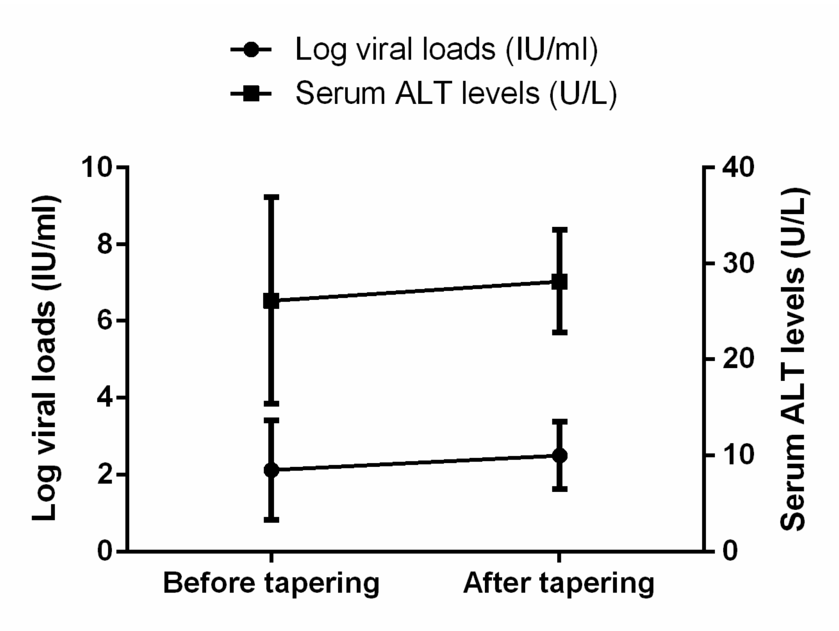

(E)

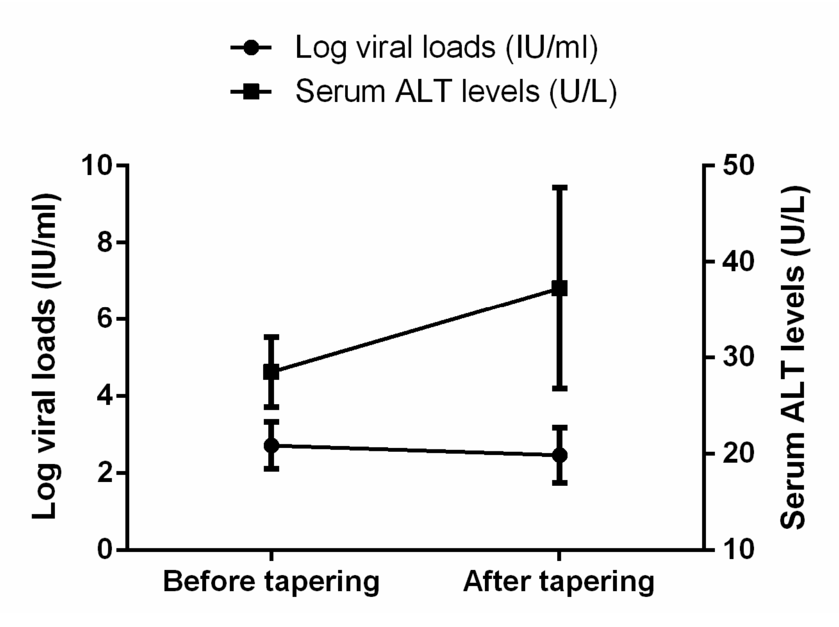

Figure S1. The change in serum levels of viral loads and ALT in RA patients with chronic hepatitis B before and after tapering of b/tsDMARDs, with respect to (A) 33 patients whose status of antiviral drugs use was consistent before and after b/tsDMARDs tapering, (B) 8 patients whose percentage decrease in b/tsDMARDs was equal to or more than 50%, (C) 12 patients who used TNF-α inhibitors, (D) 6 patients who used abatacept, and (E) 8 patients who used tofacitinib. Data are presented as mean ± SEM. ALT, alanine aminotransferase; b/tsDMARDs, biological/targeted synthetic disease-modifying antirheumatic drugs; RA, rheumatoid arthritis; TNF, tumor necrosis factor.

(A)

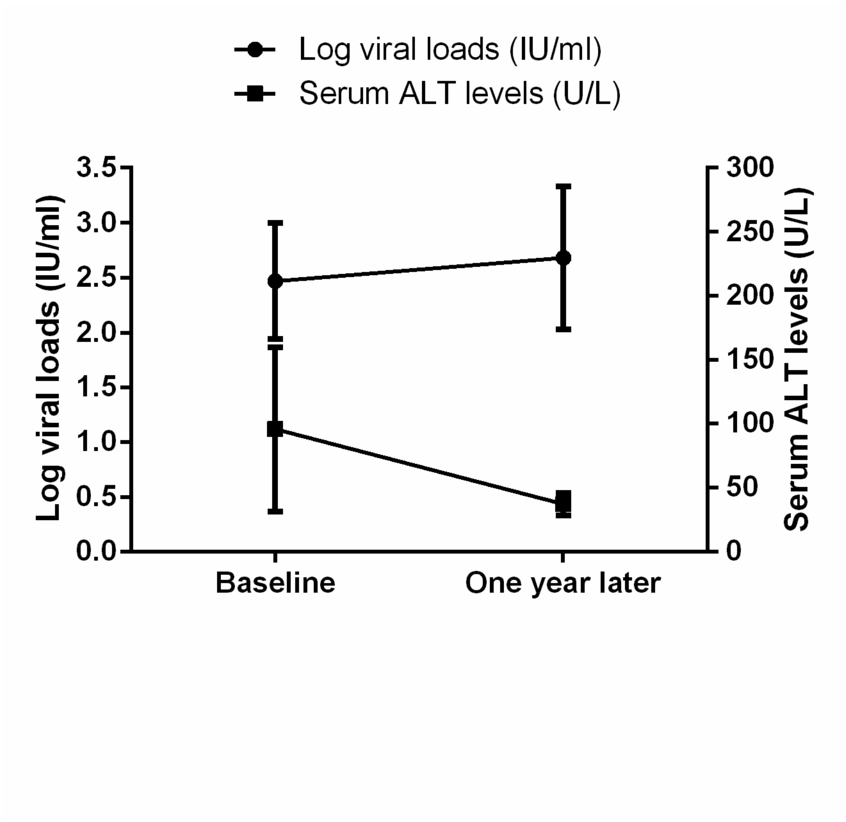

(B)

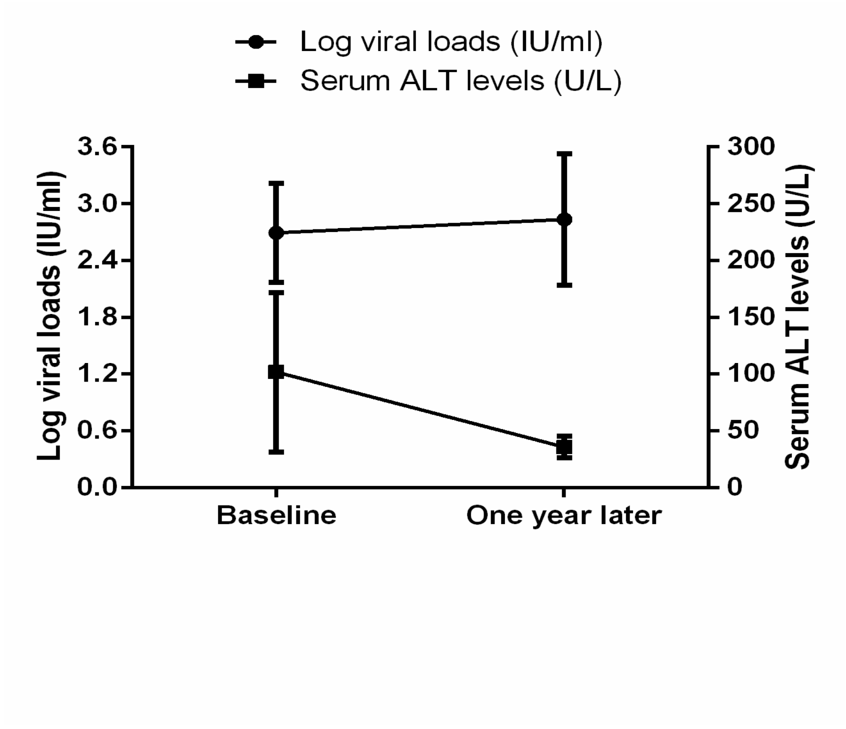

Figure S2. The change in serum levels of viral loads and ALT with time in RA patients who used b/tsDMARDs for more than 2 years without dose reduction, in regards to (A) all 12 patients, (B) 11 patients with detectable HBV DNA levels at baseline[a]. Data are presented as mean ± SEM. ALT,

alanine aminotransferase; b/tsDMARDs, biological/targeted synthetic disease-modifying antirheumatic drugs; HBV, hepatitis B virus; RA, rheumatoid arthritis; TNF, tumor necrosis factor. [a]defined as 2 years since the first prescription of b/tsDMARDs.
